# Glypican-1 Level Is Elevated in Extracellular Vesicles Released from MC38 Colon Adenocarcinoma Cells Overexpressing Snail

**DOI:** 10.3390/cells9071585

**Published:** 2020-06-30

**Authors:** Izabela Papiewska-Pająk, Damian Krzyżanowski, Maria Katela, Romain Rivet, Sylwia Michlewska, Patrycja Przygodzka, M. Anna Kowalska, Stéphane Brézillon

**Affiliations:** 1 Institute of Medical Biology, Polish Academy of Sciences, 93-232 Lodz, Poland; dkrzyzanowski@cbm.pan.pl (D.K.); pprzygodzka@cbm.pan.pl (P.P.); mkowalska@cbm.pan.pl (M.A.K.); 2 CNRS UMR 7369, Matrice Extracellulaire et Dynamique Cellulaire (MEDyC), Laboratoire de Biochimie Médicale et Biologie Moléculaire, Université de Reims Champagne Ardenne, 51100 Reims, France; catelam4ria@gmail.com (M.K.); romain.rivet@univ-reims.fr (R.R.); 3 Laboratory of Microscopic Imaging and Specialized Biological Techniques, Faculty of Biology and Environmental Protection, University of Lodz, 90-237 Lodz, Poland; sylwia.michlewska@biol.uni.lodz.pl; 4 Department of Hematology, The Children’s Hospital of Philadelphia, Philadelphia, PA 19104, USA

**Keywords:** colon adenocarcinoma, extracellular vesicles, epithelial-to-mesenchymal transition, Glypican-1

## Abstract

The transcription factor Snail triggers epithelial-to-mesenchymal transition (EMT), endowing cancer cells with invasive properties during tumor progression. Extracellular vesicles (EVs) released from cancer cells at various stages of cancer progression are known to influence the tumor pre-metastatic niche and metastatic potential. The aim of this study was to analyze the effect of Snail on murine colon adenocarcinoma cells (MC38 line) and on the characteristics of their EVs. Stable clones of Snail-overexpressing MC38 cells were investigated in vitro versus Mock cells. Increased expression of matrix metalloproteinase MMP-14 and augmented activity of MMP-9 and -14 were observed in Snail-MC38 cells. There was no change in the transcriptomic profile of proteoglycans in Snail-MC38 cells; however, the protein level of Glypican-1 (GPC1) was enhanced in EVs released from those cells. Our finding that GPC1 protein level was enhanced in EVs released from MC38 cells that overexpressed Snail and were in an early EMT stage might explain the specificity of the GPC1 biomarker in colon cancer diagnosis. Further, our data suggest that Snail, by changing the level of GPC1 on EVs released by colon cancer cells, may affect the generation of a distant premetastatic niche and metastatic organotropism in colon adenocarcinoma.

## 1. Introduction

Colorectal cancer (CRC), the third and second most commonly diagnosed cancer in men and in women, respectively, is often diagnosed in its late stage, when the cancer becomes metastatic [1]. To increase their invasive ability and to relocate to the distant places to form metastases, cancer cells undergo epithelial-to-mesenchymal transition (EMT). EMT was recognized as a foremost driver of epithelial-derived tumor malignancies [2,3,4,5]. In the EMT process, cancer cells, in response to various mechanical and chemical signals, modulate their shape and/or adhesive properties that allow them to dissociate from primary carcinomas, migrate, and disseminate to distant sites [6].

In numerous studies, metastatic cancer cells were found to express the Snail protein, a zinc-finger transcription factor that represses the transcription of E-cadherin and triggers EMT [7,8]. The impact of Snail on the characteristics of CRC cells undergoing EMT has been investigated by our group in various aspects for several years [9,10,11].

Cancer cells, like all other cells, release extracellular vesicles (EVs) that are heterogeneous vesicles of different sizes; small EVs are formed inside endosomal compartments (i.e., exosomes). They can deliver various molecules to distant places both in physiological and in pathological conditions. EVs were found to be important players in cell–cell communication but are also useful as biomarkers of stages of cancer [12]. EVs impact on cancer development is under intensive investigation [13,14,15,16], and knowledge of their properties may help in both cancer diagnosis and treatment [17]. Quantitative and qualitative changes in the proteome of EVs were reported during cancer cell transition to the mesenchymal state of human squamous cell carcinoma [18]. The presence of EVs promotes metastasis in numerous cancers [19,20,21,22]. Exosomes are also released from colon cancer cells [23]. Circulating exosomes were detected in colon cancer patients [24,25]. Our prior studies have shown that EVs released from human colon cancer cells (HT29) that undergo EMT triggered by Snail have a different miRNA expression pattern as compared to unstimulated cells [10]. Thus, when colon cancer is in a metastatic stage, EVs released from cells that undergo EMT can affect the host cells differently and also serve as biomarkers for advance stages of CRC.

Proteoglycans (PGs), that are important for the structure and function of the extracellular matrix, were found to be paramount in cancer [26]. They facilitate cell–cell and cell–matrix interactions and promote signaling that leads to tumor cell proliferation, spreading, and angiogenesis [26,27,28,29]. Chemotherapy has been shown to induce the release of exosomes enriched with heparanase, which helps to degrade the extracellular matrix and affects both cancer and host cells [30,31]. Among other proteoglycans, Glypican-1 (GPC1) has been found on the exosomes formed by pancreatic cancer cells and was suggested to be a useful indicator of the early stages of this cancer [32]. In addition, GPC1 found on patients exosomes as well as its regulative miRNAs are described as biomarkers in colon cancer [24,25]. The aim of this study was to analyze the effect of Snail-induced EMT on the characteristics and the profile of surface proteoglycans expression in EVs released from CRC cells.

## 2. Materials and Methods

### 2.1. Cell Culture and Reagents

The mouse colon adenocarcinoma cell line MC38 (originally supplied by Dr. James W. Hodge, National Cancer Institute, Bethesda, MD, USA) was cultured in RPMI 1640 medium (Thermo Fisher Scientific, Waltham, MA, USA) supplemented with 10% fetal bovine serum (FBS, BioWest, Riverside, MO, USA; VWR/Avantor, Radnor, PA, USA) and the antibiotics Penicillin-Streptomycin (Sigma-Aldrich, St. Louis, MO, USA) and primocin (Invivogen, San Diego, CA, USA) in a 90–95% humidified atmosphere of 5% CO_2_. Unless otherwise stated, all reagents were purchased from Sigma-Aldrich (St. Louis, MO, USA) or Thermo Fisher Scientific (Waltham, MA, USA).

### 2.2. MC38 Nucleofection and Generation of Stable Clones

Stable transfection of MC38 with the *Snail*-pcDNA3.1 vector was performed as described previously [33]. Two positive clones were generated through G418 selection, i.e., Snail-overexpressing MC38 clones #2 and #6 (Snail 2, Snail 6), and compared throughout the study with control pcDNA-MC38 cells (Mock).

### 2.3. Confocal Microscopy

Cells were grown until around 80% of confluence. For laser confocal microscopy, the cells were fixed in 4% paraformaldehyde. Cells were permeabilized with 0.1% Triton X-100 and incubated at 4 °C with primary antibodies, listed in Table S1, followed by secondary Alexa Fluor^®^ 488-conjugated antibodies. Alexa Fluor^®^ 568-conjugated phalloidin was used to label filamentous actin. The laser scanning microscope LSM 710 NLO (Zeiss, Oberkochen, Germany) was used.

### 2.4. Proteolytic Activities of Matrix Metalloproteinases (MMPs)

To determine MMP-2 and MMP-9 activities, MC38-clones-conditioned media were concentrated and analyzed on SDS-polyacrylamide gels containing 1 mg/mL gelatin, and the MMP-14 activity was evaluated as described earlier [33].

### 2.5. Isolation of Extracellular Vesicles from Conditioned Media

The Snail-MC38 and control clones were grown to 70–80% confluence. EV fractions were obtained after sequential centrifugations and ultracentrifugation for 1.5 h at 100,000× *g* and followed by washing in PBS and another ultracentrifugation. The final EV pellets were resuspended in PBS and stored at −80 °C until use ([10] and modified from [34]). We submitted all relevant data of our experiments to the EV-TRACK knowledgebase (EV-TRACK ID: EV190098) [35].

### 2.6. Western Immunoblotting

The cellular and EV proteins were extracted with RIPA buffer [36], and equal amounts of protein extracts (protein concentration measured by BCA assay) were subjected to SDS-PAGE analysis, transferred onto PVDF membranes, and incubated at 4 °C with specific antibodies overnight (Table S1). Specific HRP-conjugated secondary antibodies were used, and protein bands were detected using an enhanced chemiluminescence kit and Kodak BioMax Light Film (Eastman Kodak, Rochester, NY, USA).

### 2.7. RNA Isolation and Real-Time PCR Analysis

Total RNA from MC38 clones and from EVs was isolated using the miRCURY™ RNA Isolation Kit (Qiagen, Hilden, Germany) and treated with the High-Capacity cDNA Reverse transcription kit. Real-time PCR was performed using the indicated primers (Table S2) and the TaqMan Universal PCR master mix and the ABI Prism7900-HT detection system [9]. Gapdh and Eef1a1 mRNA transcripts served as internal controls. The amount of target mRNA in the various samples was estimated using the 2^−ΔCT^ relative quantification method with DataAssist v.3.01 software.

### 2.8. Transmission Electron Microscopy (TEM)

TEM assay was used to evaluate the shape and size of EVs. Samples were placed on 200-mesh copper grids with carbon surface. The samples were negatively stained with 2% uranyl acetate. Transmission electron microscopy images were obtained using JEOL-1010 (JEOL, Tokyo, Japan).

### 2.9. Size Distribution Analysis

The size distribution of the EVs was analyzed with a Litesizer™ 500 device by courtesy of the company representative (Anton Paar, Graz, Austria). EV suspensions in PBS were transferred to single-use cuvettes for dynamic light scattering (DLS) measurements and were analyzed in triplicate, averaging 30 single measurements.

### 2.10. Cell Proliferation Assay

The cells were seeded (2.5 × 10^3^ cells/200 μL/well) onto a 96-well plate; 100 μL of medium was removed from each well every other day and replaced with fresh growth medium. Cell density was measured at 0, 72, and 96 h using the Cell Counting Kit-8 (Sigma-Aldrich). The absorbance at 450 nm was measured after 2 h of incubation with CCK-8 according to the manufacturer’s instructions.

### 2.11. Statistical Analysis

Data are presented as mean ± SD or median with interquartile ranges. To confirm the Gaussian distributions of raw data, the Shapiro–Wilk’s test was used. According to normality distribution, to test the differences between two groups, Student’s *t*-test (Gaussian) or Mann–Whitney’s *U* test (non-normal) was used. To analyze the differences among group means, one-way ANOVA with appropriate post hoc multiple comparison, Dunnett’s or Tukey’s test (Gaussian), or one-way ANOVA on ranks Kruskal–Wallis *H* test (non-normal) was used.

## 3. Results

### 3.1. Characterization of Snail-MC38 Stable Clones

**Figure 1:**
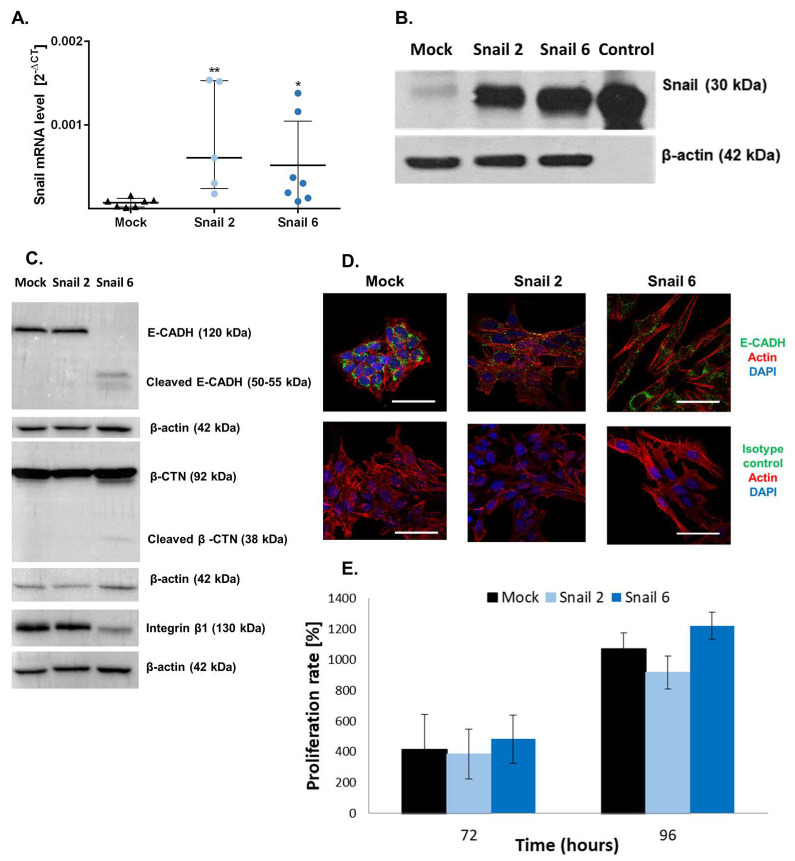
Characterization of Snail-MC38 stable clones. (**A**) Relative Snail mRNA expression in pcDNA-MC38 (Mock) and Snail-MC38 clones (clone #2 and #6: Snail 2 and Snail 6, respectively). Snail mRNA levels were normalized to *GAPDH* and *EEF1A1*. Results are shown as median with interquartile range; * *p* ≤ 0.05 and ** *p* ≤ 0.01, *n* = 7. (**B**) Representative Western blot analysis of Snail protein expression in Mock and Snail-MC38 clones. Control: recombinant human Snail. (**C**) Western blot analysis of E-cadherin (E-CADH), β-catenin (β-CTN), integrin β1, and β-actin in Mock and in Snail-overexpressing MC38 cells. (**D**) Distribution of E-CADH and actin in Mock and Snail-overexpressing MC38 cells (representative images, scale bar: 50 µm). (**E**) Cell proliferation rate of Mock- and Snail-MC38 clones at 72 and 96 h. Data are shown as mean ± SEM. Uncropped gels from Figure 1B,C and densitometry of Western blot results are shown in supplementary Figure S1B and Figure S1C, respectively.

The levels of Snail expression were evaluated in pcDNA-MC38 (Mock) and Snail-overexpressing (Snail-MC38) clones #2 and #6 by real-time PCR and Western blot analyses (Figure 1A,B). E-cadherin (E-CADH) and integrin β1 expression levels were decreased in Snail-MC38 clone #6. Increase of β-catenin (β-CTN) expression in that clone was also observed (Figure 1C). Further, as presented in Figure 1D, Snail-MC38 cells acquired a spindle and dendritic shape, and cell-to-cell contacts became loose. We also performed a proliferation assay comparing Snail-MC38 clones with Mock cells (Figure 1D). No significant differences were detected.

### 3.2. Effect of Snail Overexpression in MC38 Cells on MMPs Activities and Gene Expression of Proteoglycans

**Figure 2:**
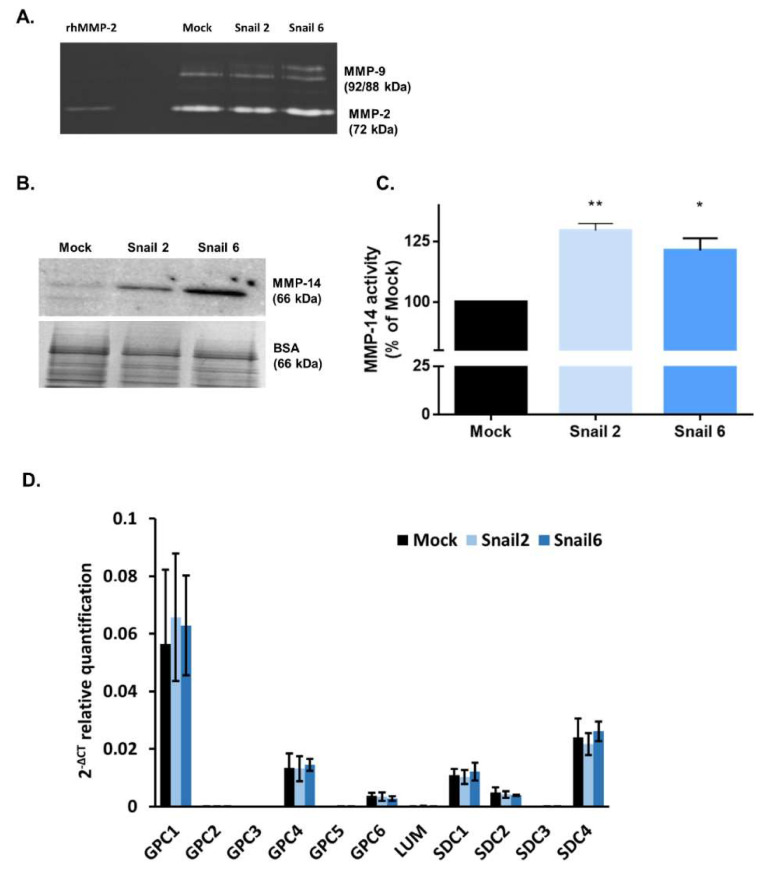
Effect of Snail on matrix metalloproteinase (MMP)-2, -9, and -14 activity and proteoglycan gene expression in MC38 cells. (**A**) Zymography analysis for MMP-2 and MMP-9 activity in Mock and in Snail-MC38 clones. Control: recombinant human MMP-2. (**B**) Western blot analysis of MMP-14 protein expression in Mock and in Snail-MC38 clones. The total protein normalization (TPN) method was used. (**C**) MMP-14 activity in Snail-overexpressing MC38 clones expressed as percentage of Mock, * *p* < 0.05, ** *p* < 0.01. Data are shown as median with interquartile range. (**D**) Real-time RT-PCR analysis of proteoglycans expression in Mock and Snail-overexpressing MC38 cells; *n* ≥ 3.

As presented in Figure 2A, in mouse MC38 cells, Snail upregulated the level of gelatinase pro-MMP-9, while the level of MMP-2 was not affected, as evaluated by gelatin zymography.

The level of MMP-14 protein (Figure 2B) and its activity (Figure 2C) was increased in both Snail-MC38 clones. Potentiation of MMP-14 activity by Snail was previously observed by us in B16F1 melanoma cells, and this effect was inhibited by Lumican (LUM) [33]. In contrast to Snail-B16F1 cells, the increased MMP-14 activity in Snail-MC38 cells was not affected by the presence of LUM (data not shown). This result led us to investigate whether LUM was endogenously expressed in Mock- or Snail-MC38 cells. As shown in Figure 2D, *Lum* gene expression was barely detectable by RT-PCR and equal in all MC38 clones. In both Mock- and Snail-MC38 clones, we also analyzed gene expression of other proteoglycans that are known to impact cancer progression [37]. *Gpc1* and syndecan-4 (*Sdc4*) were the two proteoglycans with the most elevated gene expression in MC38 cells, but the overexpression of Snail did not exert any effect (Figure 2D).

### 3.3. Characterization of EVs Derived from MC38 Cells: PG Expression Profile

**Figure 3:**
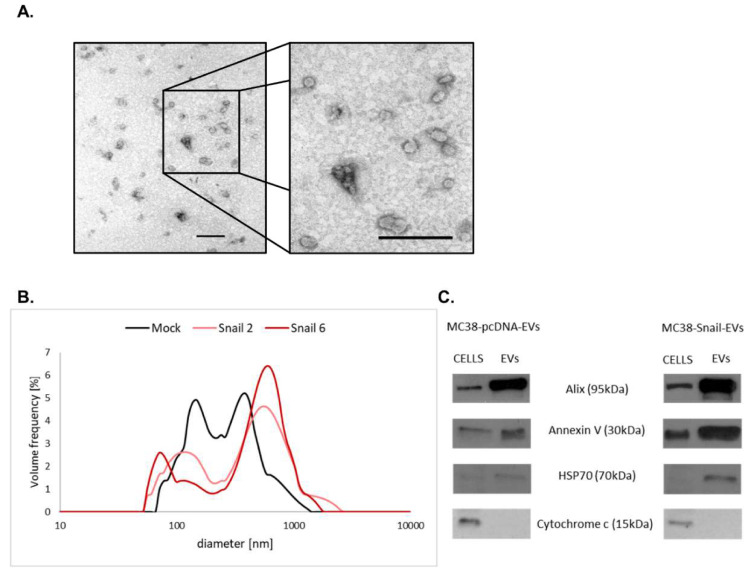
Characterization of pcDNA-MC38- and Snail-MC38-derived extracellular vesicles (EVs). (**A**) Representative electron microscopic images of EVs derived from Snail-MC38 cells (Scale bars: 200 nm). (**B**) Average size distribution of EVs measured by dynamic light scattering (DLS). (**C**) Representative Western blot analysis of EV markers expression in pcDNA- and Snail-MC38 cells and corresponding EVs. Immunoblotting demonstrates the enrichment of Alix and Annexin V, the presence of HSP70, and the absence of Cytochrome C in EVs compared with cell lysates. For both cells and EVs samples, the same amount of proteins per lane was loaded (10 μg for Alix, Annexin V, and HSP70 and 20 μg for Cytochrome c).

We also examined the expression of proteoglycans in EVs released from Mock- and Snail-MC38 cells. Isolated EVs were first checked for their purity (Figure 3A) and size distribution (Figure 3B).

There was no significant difference in the characteristics of EVs released from Mock- or Snail-MC38 cells; however, we observed that the size of EVs released from Snail-MC38 cells was more heterogenous. EVs released from cells overexpressing Snail were slightly smaller (60 nm) or bigger (700 nm) in comparison to those released by mock cells (150 nm or 400 nm, respectively). We also characterized EVs for the expression of their specific markers in comparison with cell extracts (Figure 3C). As expected, EVs exhibited increased expression of Alix, Annexin V, and HSP70, but not Cytochrome c.

EVs are a heterogenous group of phospholipid-bilayer particles present in all body fluids. They can be divided into three main subpopulations including exosomes (exo), microvesicles/ectosomes (MVs), and apoptotic bodies (APOs), which are different in size, formation process, and content. Intestinal epithelial adenocarcinoma cell lines produce fewer exosomes compared to other cancer types, thus we decided to investigate the biology of EVs (pooled exo and MVs) released from CRC cells in the early EMT process. In the organism during cancer progression, both exo and MVs are released from non-apoptotic cells.

**Figure 4:**
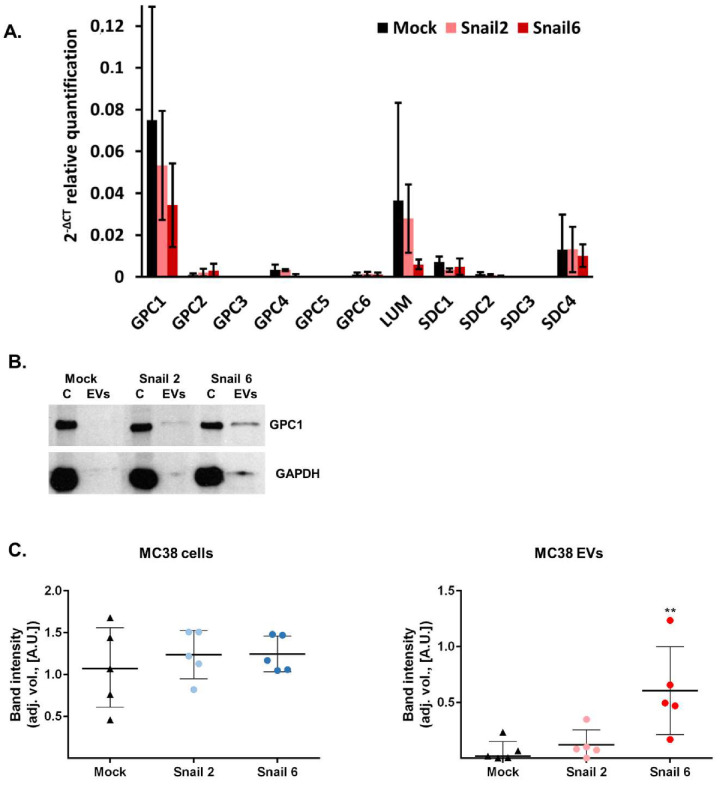
Proteoglycans gene expression in EVs of Mock and Snail-overexpressing MC38 cells. (**A**) Real-time RT-PCR analysis of proteoglycans expression in Mock-MC38- and Snail-MC38-derived EVs; *n* ≥ 3. (**B**) Western blot analysis of Glypican-1 (GPC1) expression in cell lysates (C) from Mock- and Snail-MC38 cells and corresponding EVs. (**C**) Densitometry analysis of protein bands. For both MC38 cells and EVs: adjusted volume (adj. vol.) of GPC1 band was quantified by ImageLab^®^ from Biorad (*n* = 4, ** *p* < 0.01).

As detected by real-time RT-PCR analysis, proteoglycan gene expression in EVs released from Mock- and Snail-MC38 cells (Figure 4A) was similar. As observed in MC38 cells (Figure 2D), *Gpc1* was again the major glypican found in EVs. *Sdc4* and *Lum* RNAs were also detectable (Figure 4A).

While there was no increase in GPC1 RNA in EVs obtained from Snail-MC38 cells, an increase of the GPC1 protein level was observed in the EVs lysates of both Snail-MC38 clones, as calculated by comparison of the protein bands intensities in x-ray images from ECL–western immunoblotting (Figure 4B,C). GPC1 core protein was hardly detectable in Mock-MC38 EVs, but its amount was around two times higher in EVs released from clone Snail 2 and even more than nine times higher for clone Snail 6. The increase of GPC1 level in Snail-MC38 clone #6 was statistically significant.

## 4. Discussion

Our previous studies showed that Snail, an early regulator of EMT, affects human HT29 CRC cells transcriptome and miRNA profile and changes HT29 cells phenotype to a promigratory one [9,10]. In this paper, we described Snail effect on mouse CRC line MC38 in order to characterize it before moving to in vivo mouse models of colon cancer. In this work, we studied the effect of Snail overexpression on the profile of surface proteoglycans expression in EVs released from MC38 cells. We are aware of the fact that we limited this study to only one cell line. Two clones with different levels of Snail overexpression were used. However, only Snail-MC38 clone #6 showed characteristics that indicate EMT, which is known to be associated with tumor progression and metastasis. This result is consistent with our previous studies on the effect of Snail on human HT-29 CRC and B16F1 melanoma cells [9,33].

We showed earlier that Snail upregulates the activities of MMP-9 and MMP-14 in B16F1 melanoma cells [33]. The same was reported here for MC38 cells. MMPs are known to be major key regulators of the extracellular matrix (ECM) remodeling and play a role in facilitating the invasion of cancer cells undergoing EMT [38]. We also showed previously that extracellularly added Lumican (LUM), that belongs to the family of PGs, inhibits MMP-14 activity induced by Snail in B16F1 melanoma cells [33]. However, no effect of LUM was observed previously by us in human HT-29 cells and now in mouse MC38 colon cancer cell lines. These findings suggest that the effect of LUM is specific for certain cancer cells and that LUM may be a candidate as an inhibitor of MMP-14 and perhaps cancer cell invasion in the case of melanoma, but not for colon adenocarcinoma cells [30,33,39,40,41].

PGs are major macromolecules of the extracellular matrix accumulating in remodeled stroma [26]. They are classified according to their localization as ECM-associated, pericellular, cell surface-associated, and intracellular. Extracellular PGs include small leucine-rich PGs such as LUM [26]. The cell surface proteoglycans include syndecans and glypicans. The expression of these PGs is markedly affected during cancer progression in tumor and stromal cells [42]. They are key regulators of cell–cell and cell–matrix interactions, affecting downstream signaling pathways involved in tumor cell proliferation, spreading, and angiogenesis [26].

In our hands, *Gpc1* was found to be the major glypican expressed in MC38 cells, and the overexpression of Snail did not have any effect on the gene expression of this proteoglycan. However, interestingly, while there was no change in the *Gpc1* RNA in EVs released from Snail-MC38 cells, as compared to mock cells, an increased amount of the GPC1 core protein in the lysates of EVs released from Snail-MC38 clones was observed. Whether the increased amount of the GPC1 protein is present in bigger or smaller EVs remains to be examined. The high and statistically significant increase was observed in EVs released from Snail-MC38 clone #6 and seems to be characteristic of EVs released from cells that underwent an early EMT process.

It was shown earlier that GPC1 accumulates in pancreatic cancer cell-derived exosomes [32]. The level of circulating GPC1-positive exosomes increases significantly and correlates with survival in pre- and post-surgical patients [32]. Thus, GPC1-positive exosomes emerged as a potential non-invasive diagnostic and screening tool to detect early stages of this disease [32]. GPC1 was suggested to be a biomarker of relapse of stage III colorectal cancer, and its level was correlated with poor survival [24,25].

## 5. Conclusions

Our finding that GPC1 protein level was enhanced in EVs released from MC38 cells that were in an early EMT stage might explain, at least in part, the specificity of the GPC1 biomarker in colon cancer diagnosis. Further, our data suggest that Snail, by changing the level of GPC1 on EVs released by colon cancer cells, may affect the generation of a distant premetastatic niche and metastatic organotropism in colon adenocarcinoma.

## Supplementary Materials

The following are available online at https://www.mdpi.com/2073-4409/9/7/1585/s1, Table S1: List of antibodies used for immunocytochemistry and western immunoblotting; Table S2: List of primers used for real-time PCR; Figure S1B: Original representative full-length blots of Figure 1B showing Snail protein detection and densitometry plots. Recombinant human Snail (positive control) was added in excess to be more visible; Figure S1C: Original full-length blots of Figure 1C and densitometry plots; Figure S2B: Original full-length blots of Figure 2B showing MMP-14 expression. The wild-type MC38 cells’ track was cropped in Figure 2B since it was similar to that of Mock-MC38 cells; Figure S4B: Original full-length blots of Figure 4B showing GPC1 expression. Each of them were taken into account while densitometry analysis was performed; Figure S3: Amount of EVs released by Mock and Snail-overexpressing MC38 clones shown as µg of protein in EV lysates obtained from 10 × 10^6^ cells.

## Abbreviations

CRC	Colorectal cancer
EMT	Epithelial-to-mesenchymal transition
EVs	Extracellular vesicles
GPC	Glypican
MMPs	Matrix Metalloproteinases
PGs	Proteoglycans
SDC	Syndecan
